# Supplementary figures and images for: Berry Consumption and Sleep in the Adult US General Population: Results from the National Health and Nutrition Examination Survey 2005–2018
Source: Nutrients. 2023 Dec 15;15(24):5115. doi: 10.3390/nu15245115 (PMC10745662; doi:10.3390/nu15245115)

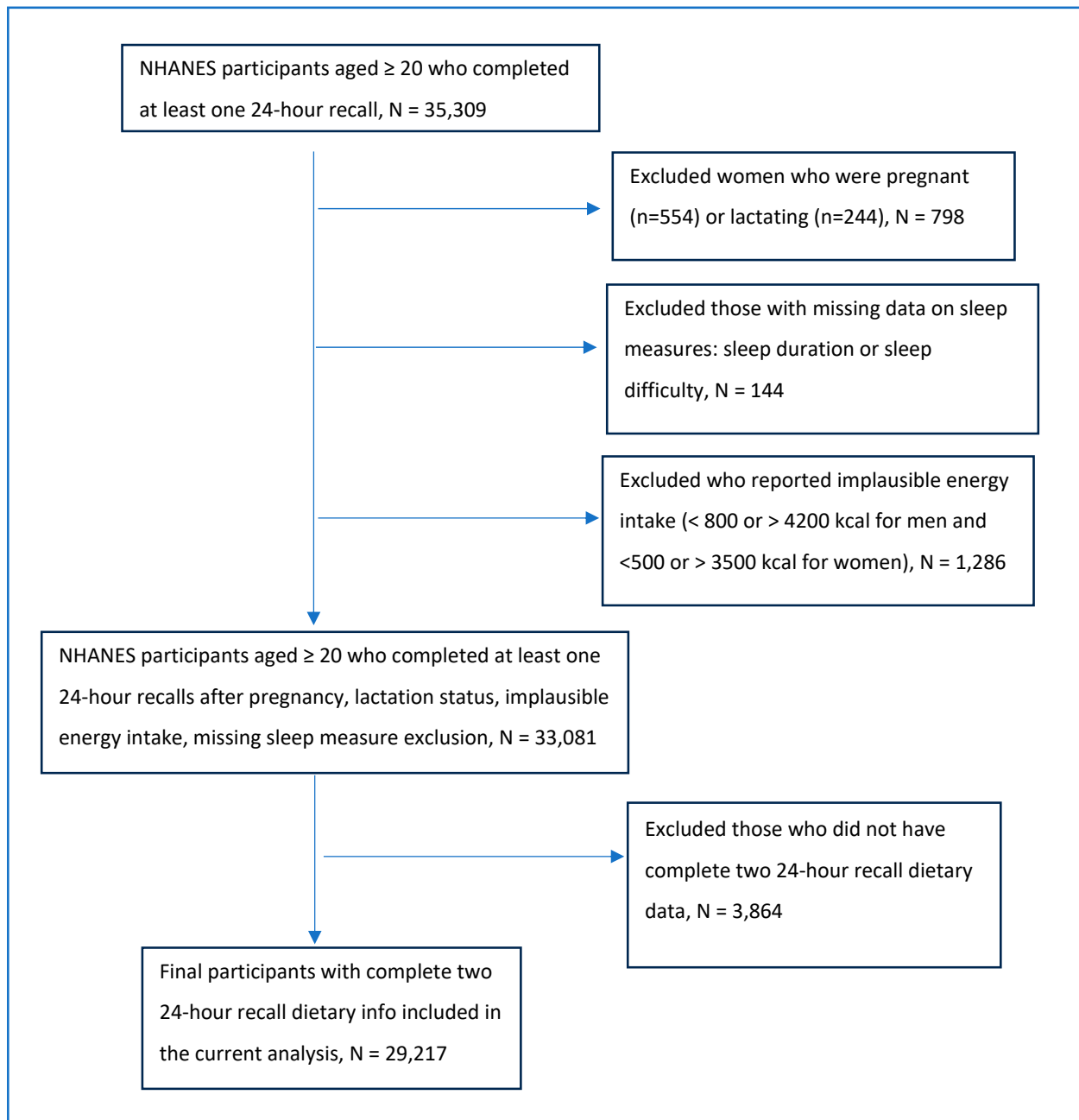

**Figure S1.** Participant flow chart

Supplement: Supplementary file 1 [file nutrients-15-05115-s001.zip › nutrients-2755701-supplementary.pdf]
